# Long-term evaluation of pediatric ACL reconstruction: high risk of further surgery but a restrictive postoperative management was related to a lower revision rate

**DOI:** 10.1007/s00402-021-04135-0

**Published:** 2021-08-30

**Authors:** Frida Hansson, Eva Bengtsson Moström, Magnus Forssblad, Anders Stålman, Per-Mats Janarv

**Affiliations:** 1 Department of Molecular Medicine and Surgery, Stockholm Sports Trauma Research Center, Karolinska Institutet, Stockholm, Sweden; 2 Capio Artro Clinic, FIFA Medical Centre of Excellence, Sophiahemmet Hospital, Valhallavägen 91, 11486 Stockholm, Sweden

**Keywords:** ACL reconstruction, Revision, Children, Adolescents, Postoperative bracing

## Abstract

**Introduction:**

The guidelines regarding rehabilitation after pediatric anterior cruciate ligament reconstruction (ACLR) are sparse. The aim of the study was to retrospectively describe the long-term outcome regarding further surgery and with special emphasis on the revision rate after two different postoperative rehabilitation programs following pediatric ACLR.

**Material and methods:**

193 consecutive patients < 15 years of age who had undergone ACLR at two centers, A (*n* = 116) and B (*n* = 77), in 2006–2010 were identified. Postoperative rehabilitation protocol at A: a brace locked in 30° of flexion with partial weight bearing for 3 weeks followed by another 3 weeks in the brace with limited range of motion 10°–90° and full weight bearing; return to sports after a minimum of 9 months. B: immediate free range of motion and weight bearing as tolerated; return to sports after a minimum of 6 months.

The mean follow-up time was 6.9 (range 5–9) years. The mean age at ACLR was 13.2 years (range 7–14) years.

The primary outcome measurement in the statistical analysis was the occurrence of revision. Multivariable logistic regression analysis was performed to investigate five potential risk factors: surgical center, sex, age at ACLR, time from injury to ACLR and graft diameter.

**Results:**

Thirty-three percent had further surgery in the operated knee including a revision rate of 12%. Twelve percent underwent ACLR in the contralateral knee. The only significant variable in the statistical analysis according to the multivariable logistic regression analysis was surgical center (*p* = 0.019). Eight percent of the patients at center A and 19% of the patients at B underwent ACL revision.

**Conclusions:**

Further surgery in the operated knee could be expected in one third of the cases including a revision rate of 12%. The study also disclosed a similar rate of contralateral ACLR at 12%. The revision rate following pediatric ACLR was lower in a center which applied a more restrictive rehabilitation protocol.

**Level of evidence:**

Case-control study, Level III.

## Introduction

### Background

The incidence of anterior cruciate ligament (ACL) injuries has increased among children and adolescents and is higher than in adults [1, 2]. Higher participation rates in competitive youth sports, early specialization and increased intensity at young age have been suggested as reasons for the increase in ACL injuries, but the scientific evidence level is low [3]. It was suggested that skeletally immature children are more vulnerable to injury for several reasons, such as relative changes in joint forces during rapid bone growth and the difference between physical and cognitive development in early adolescence leading to a behavior of higher risk taking [4]. Several studies have also confirmed that sex plays an important role. Females were significantly more vulnerable for an ACL injury and the incidence was in some studies more than twice as high compared with males [5]. However, these large sex differences has not been disclosed regarding graft ruptures and revision rates [6].

Non-operative or delayed anterior cruciate ligament reconstruction (ACLR) in this group of patients has previously been preferred, due to the risk of growth disturbances. In a recent Norwegian long-term follow-up by Ekås et al., ACL injured patients younger than 13 years of age were treated with a structured rehabilitation program. The non-operative treatment was successful in half of the patients, but only a few returned to their pre-injury activity level. A delayed ACLR was performed in the rest of the patients, due mainly to instability [7]. It must be taken into account that there is an increased risk of meniscal and cartilage injuries with time from injury to surgery, and thus ACLR is considered necessary in children and adolescents with an unstable knee at the desired activity level [8, 9]. Improved knee function in patients who have undergone early reconstruction has been reported in several studies [10].

One severe complication after ACLR is a rupture of the graft. The risk was significantly higher in the pediatric population compared with adults [11, 12]. A smaller graft size may contribute to a higher risk of graft rupture. Snaebjörnsson et al. found a correlation in adults between graft diameter and revision rates [13], but this has not yet been clarified in the pediatric population.

To achieve a good outcome following surgery, optimizing postoperative management is of great importance [14]. The guidelines on rehabilitation after pediatric ACLR are sparse. In adults, there was no scientific support for using a postoperative brace in order to reduce complications or re-injury rates [15–17]. The efficiency of postoperative bracing is unclear in a pediatric population, but it has been suggested that a brace might provide both physical and psychological support. The role of accelerated rehab and postoperative bracing needs to be further elucidated in pediatric patients to aid the development of more optimized postoperative guidelines in this population [18, 19].

### Objectives

The aim of the study was to retrospectively describe the long-term outcome after ACLR in patients under 15 years of age regarding further surgery in both the ACL reconstructed knee and contralateral knee, and with special emphasis on the results after two different postoperative rehabilitation programs with focus on revision rate, and taking into account sex, age at ACLR, time from injury to ACLR, and graft diameter. We hypothesized that a more restrictive postoperative rehabilitation protocol would lead to a lower revision rate.

## Materials and methods

### Treatment

The indication for ACLR was instability at desired activity level or a wish to return to pre-injury activity level with a high exposure to pivoting activities. Patients with meniscal injuries suitable for repair were also recommended an ACLR. At both centers, arthroscopic ACLR with transphyseal drilling was performed and hamstring autografts from the ipsilateral leg were used. The femur tunnel was placed with a transtibial technique. The operations at both centers were performed by experienced arthroscopic knee surgeons doing more than 30 ACLR per year and with special interest in pediatric ACLR. The minimum graft length in the tunnels, both femoral and tibial, was 2 cm in order to make sure that the graft crosses the physes, thus avoiding transphyseal solid bone bridge formation across the physes and the risk of growth disturbances. An Endobutton (Smith&Nephew, Watford, United Kingdom) was used for femoral fixation. An RCI screw (Smith and Nephew) placed distal to the physis was used for tibial fixation at A, while at B an AO bicortical screw with a washer (Smith and Nephew) was used as a post.

### Participants and data collection

All patients under the age of 15 years, with a Swedish personal ID number, who in 2006–2010 underwent ACLR at two large orthopedic centers in Sweden, A and B, were identified through the Swedish National Knee Ligament Registry (SNKLR). This registry is a national database designed to evaluate the surgical treatment of patients with an ACL injury. More than 90% of all ACLRs in Sweden are registered [20]. The ACL injury was diagnosed with MRI.

Baseline data regarding sex, age at injury and ACLR, cause of injury, surgical details, associated injuries, re-operations, and revisions, as well as follow-up data relating to the Knee and Osteoarthritis Outcome Score (KOOS) and EuroQoL-5 Dimension Questionnaire (EQ5D), were collected from the registry. In cases where bilateral ACLR was performed during the study period, the first operated knee was included in the study. The patients who had not answered the KOOS and EQ5D were contacted by post (December 2015) as a reminder to fill out the forms digitally in the SNKLR.

In addition to the registry, all the patients’ knee-specific medical contacts were identified through the Swedish National Board of Health and Welfare. The patients were identified by their unique personal ID number, which was used in every medical contact nationwide.

### Postoperative management

The two centers had different postoperative rehabilitation protocols. At A, a brace (Camp ROM Vender knee brace, CAMP Scandinavia Inc, Helsingborg, Sweden) was applied and limited weight bearing (WB) and range of motion (ROM) as follows: week 1–3: brace locked in 30° of flexion and partial WB; week 4–6: ROM 10°–90° and full WB. Return to sports was allowed after a minimum of 9 months after surgery, provided that the rehabilitation program was completed, including a one-leg hop for distance which should reach 90% of the uninjured side. The knee should have full ROM and no swelling or pain.

At B, immediate free ROM and WB as tolerated were applied after ACLR. Return to sports was allowed at a minimum of 6 months after surgery if the criteria described above were fulfilled. The criteria were assessed, as in center A, by the patient’s surgeon or physiotherapist.

### Outcome

The primary outcome measurement in the statistical analysis was the occurrence of revision. Secondary outcomes were description of any further surgery in the ACL reconstructed (for example diagnostic arthroscopy, meniscal surgery, chondral surgery or extraction of tibial post) or ACLR in the contralateral knee and subjective knee function reported in the KOOS and EQ5D at 1, 2 and 5 years after ACLR. The KOOS is divided into five subscales: Pain, Knee-related Symptoms, Activities of Daily Living (ADL), Sport and Recreation and Knee-related Quality of Life (QoL) [21]. The EQ5D is a generic health status score which includes a descriptive system questionnaire, which is calculated as the EQ5D index and a visual analog scale (EQ5D VAS) [22].

Missing data in the KOOS questionnaire were addressed according to the KOOS user guide 1.1 [23].

### Statistical methods

The SPSS 25.0 statistical software package for Mac (IBM) was used to perform the statistical analyses. The level of significance was set at *p* ≤ 0.05. The Shapiro–Wilk test of normality was conducted and non-parametric tests (the Mann–Whitney *U* test and the Chi-square test) were used when comparing the groups, while correlations were tested with Spearman’s rho correlation.

Multivariable logistic regression analysis (stepwise forward method with an inclusion criterion of 5%) was performed to investigate the relationship between five potential and dichotomized risk factors: surgical center (A/B), sex (female/male), age at ACLR above 13 years (yes/no), time from injury to ACLR more than 5 months (yes/no), and graft diameter smaller than 8 mm (yes/no). The dichotomization of age, time from injury to surgery, and graft diameter was based on the median values. The dependent variable was the occurrence of revision.

## Results

### Surgical outcome

The median time from ACL injury to ACLR was 7 months at A and 5 months at B and the difference was statistically significant (*p* = 0.002). Center A reported a higher number of meniscal injuries and a higher percentage of meniscal repairs compared with partial resections (Table 1).

**Table 1:** Patient characteristics and baseline data

Variables	Total (*n* = 193)	A (*n* = 116)	B (*n* = 77)	*p* value
Mean age at injury, years	12.5 ± 1.6 (7–14)	12.1 ± 1.7 (7–14)	13.1 ± 1.1 (8–14)	
Median age at injury, years	13 (12–14)	13 (11–13)	14 (13–14)	*p* < 0.001
Mean age at ACLR, years	13.2 ± 1.3 (7–14)	12.8 ± 1.5 (7–14)	13.6 ± 0.7 (10–14)	
Median age at ACLR, years	14 (13–14)	13 (12–14)	14 (13–14)	*p* < 0.001
Female, *n* (%)	118 (61)	62 (53)	56 (73)	*p* = 0.007
Mean time from injury to ACLR, months	7.8 ± 7.6 (0–60)	8.9 ± 7.6 (0–46)	6.3 ± 7.3 (1–60)	
Median time from injury to ACLR, months	6 (3–10)	7 (4–11)	5 (2–8)	*p* = 0.002
Operated within 1 month (%)	5 (3)	5 (4)	0 (0)	n.s
Mean graft diameter, mm	8.1 ± 0.8 (6–10)	8.2 ± 0.8 (6–10)	7.9 ± 0.7 (6–10)	*p* = 0.005
Antibiotics preop (%)	193 (100)	116 (100)	77 (100)	n.s
Cloxacillin	189	113	76	
Clindamycin	4	3	1	
Injured lateral meniscus, *n* (%)	60 (31)	47 (41)	13 (17)	*p* = 0.001
Lateral meniscal repair *n* (%)	26 (43)	25 (53)	1 (8)	
Partial resection lateral meniscus, *n* (%)	15 (25)	8 (17)	7 (54)	
No lateral meniscal surgery/undefined, *n* (%)	19(32)	14 (30)	5 (38)	
Injured medial meniscus, MM, *n* (%)	49 (25)	41 (35)	8 (10)	*p* < 0.001
Medial meniscal repair, *n* (% of injured MM)	32 (65)	30 (73)	2 (25)	
Partial resection medial meniscus, *n* (% of injured MM)	10 (20)	6 (15)	4 (50)	
No medial meniscal surgery/undefined, *n* (%)	7 (14)	5 (12)	2 (25)	
Injured MCL, *n* (%)	3 (2)	1 (1)	2 (3)	n.s
MCL surgery at time of ACL reconstruction, *n*	1	1	0	
Injured LCL, *n* (%)	0 (0)	0 (0)	0 (0)	n.s
Chondral injury, *n* (%)	3 (2)	1 (1)	2 (3)	n.s
Chondral surgery, *n*	1	0	1	

Values are expressed as mean ± SD (minimum–maximum) or as median (25th–75th percentile) when not normally distributed

Further surgery during the follow-up period appeared in one third of the ACLR knees (Table 2). Twelve percent had an ACL revision and 21% had surgery due to meniscal lesions, chondral injuries or removal of the tibial post. Twenty-three patients (12%) underwent ACLR in the contralateral knee.

**Table 2:** Follow-up minimum 5 years

Variables	Total (*n* = 193)	A (*n* = 116)	B (*n* = 77)	*p* value
Mean follow-up time, years	6.9 ± 1.4 (5–9)	6.9 ± 1.5 (5–9)	6.9 ± 1.4 (5–9)	n.s
ACL revisions (%)	24 (12)	9 (8)	15 (19)	0.016
Mean time from ACLR to revision, years	3.0 ± 2.3 (0–9)	3.9 ± 2.8 (0–9)	2.4 ± 1.8 (0–6)	n.s
Patients undergoing any re-operation after ACLR except ACLR revision	40 (21)	22 (19)	18 (23)	n.s
At least one meniscal surgery after ACLR	26 (65)	16 (73)	10 (56)	
At least one chondral surgery after ACLR	6 (15)	6 (27)	0 (0)	
Extraction of tibial post	6 (15)	0 (0)	6 (33)	
Infection	1 (0.5)	0 (0)	1 (1)	n.s
ACL reconstruction of the contralateral knee (%)	23 (12)	14 (12)	9 (12)	n.s

Values are expressed as mean ± SD (minimum–maximum) or as *n* (%)

Both centers had a 100% coverage of prophylactic preoperative antibiotics and cloxacillin was the standard choice. A postoperative infection after ACLR was seen in one patient at B and in no patients from A (Table 2). In the reported case, one arthroscopic irrigation was performed. The tissue culture showed coagulase negative staphylococcus aureus and proprioni bacteria. The patient was successfully treated with clindamycin and no further irrigation was needed.

### Patient characteristics and patient reported outcome measures

**Fig. 1:**
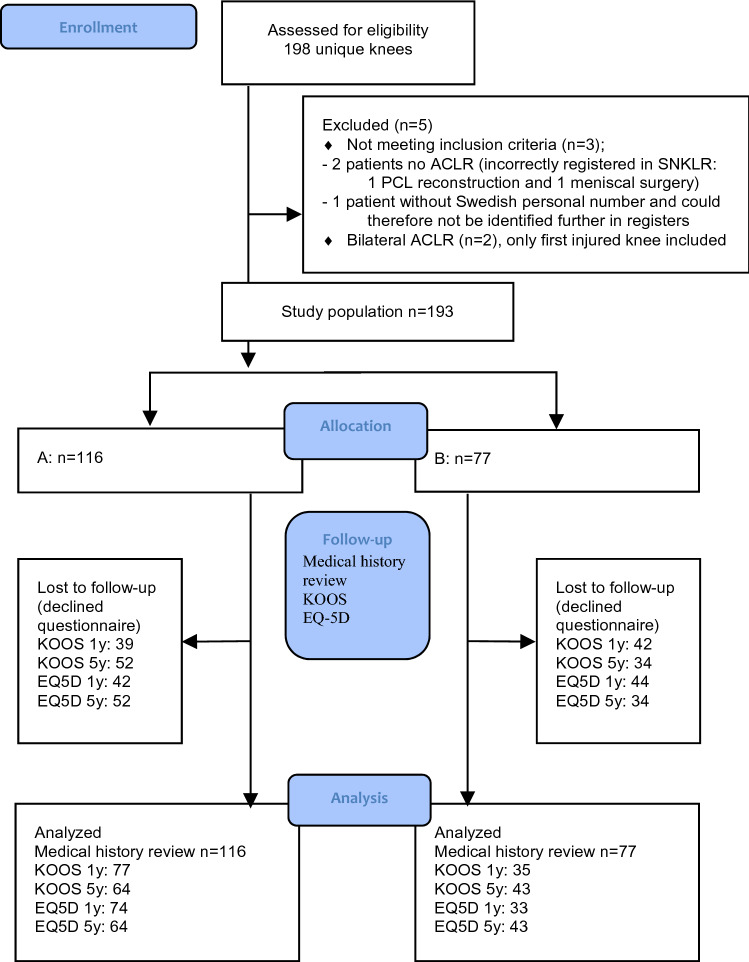
Flow diagram

One hundred and ninety-three patients met the inclusion criteria and were analyzed in the study. The patient flow chart is shown in Fig. 1.

The female:male ratio was not equally distributed between the centers. Center B had a significantly higher ratio of females (*p* < 0.007). The patients at A had a significantly lower median age at the time of index surgery compared with the patients at B (*p* < 0.001) (Table 1). The number of very young patients (< 10 years) at the time of ACLR was six at A (one 7-years-old and five 9-years-old) and none at B.

Skeletal immaturity was seen on knee MRI in 172 patients (89%). Closed physes were seen in nine patients and MRI was no longer available in 12 patients at the time of the study.

**Fig. 2:**
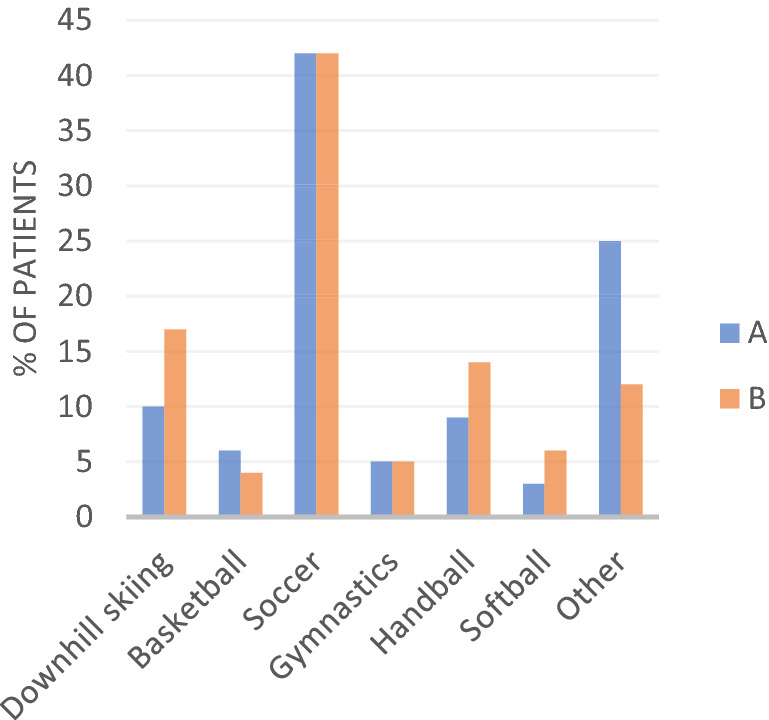
Activity at ACL injury 42% of the patients at both A and B injured their ACL during soccer

The most common cause of injury was the same at the two centers, 42% of the patients at both A and B injured their ACL during soccer (Fig. 2). The most common cause of graft rupture was soccer in Group A: 44% (4 of 9). In Group B it was soccer in 20% (3 of 15) and downhill skiing in 20% (3 of 15). In three cases (two treated at B and one at A), no history of trauma occurred prior to graft failure and the date of re-rupture was estimated from the medical history, and it was therefore vague. In two cases, the date of the trauma causing graft rupture was not exact and the date was set as the first day of the month estimated by the information in the medical records.

**Fig. 3:**
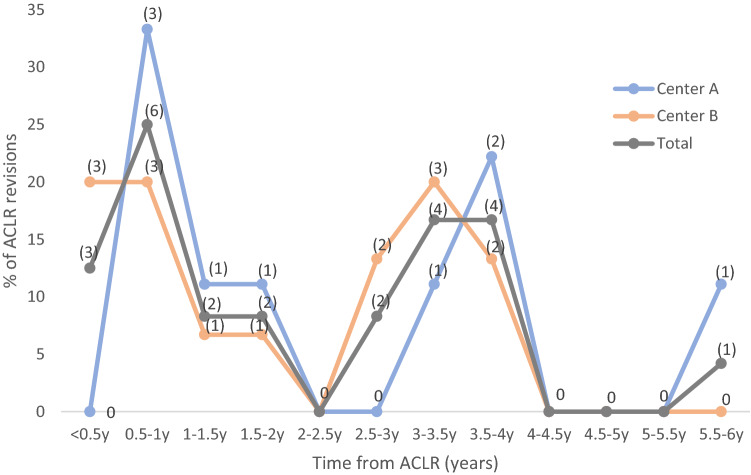
Time from ACLR to re-rupture of graft. Absolute number (*n*) of re-ruptures indicated at each time interval

Most re-ruptures occurred within the first year or after 3–4 years following ACLR. Thirty-three percent of the revised patients treated at A and 40% at B suffered their re-rupture within the first postoperative year (Fig. 3). Mean time from ACLR to re-rupture was 28 months at A and 23 months at B, without significant difference between the two centers.

No statistical difference between the two centers was seen in any of the subscales on the KOOS or EQ5D at 1, 2 and 5 years after ACLR. Values at 1 and 5 years are presented in Table 3.

**Table 3:** KOOS and EQ5D 1 and 5 years after ACLR

	Total			A			B			*p* value
	*n*	Median (25th–75th percentile)	Mean ± SD	*n*	Median (25th–75th percentile)	Mean ± SD	*n*	Median (25th–75th percentile)	Mean ± SD	
KOOS										
Symptoms 1 y	112	89 (79–89)	85.7 ± 15.1	77	89 (77–96)	85.3 ± 13.5	35	96 (82–100)	86.6 ± 18.3	n.s
Symptoms 5 y	86	86 (68–93)	81.3 ± 16.1	64	89 (68–93)	82.4 ± 16.1	43	82 (68–93)	79.7 ± 16.0	n.s
Pain 1 y	112	97 (89–100)	92.5 ± 10.2	77	97 (88–100)	92.4 ± 10.0	35	97 (89–100)	92.7 ± 10.6	n.s
Pain 5 y	107	94(83–100)	88.5 ± 14.3	64	94 (89–100)	88.9 ± 16.5	43	89 (81–97)	87.9 ± 10.1	n.s
Adl 1 y	112	100 (100–100)	97.1 ± 7.5	77	100 (99–100)	96.9 ± 7.7	35	100 (99–100)	97.5 ± 7.2	n.s
Adl 5 y	107	99 (94–100)	94.7 ± 11.8	64	100 (97–100)	94.4 ± 14.4	43	99 (93–100)	95.2 ± 6.3	n.s
Sport/play 1 y	112	90 (90–95)	82.7 ± 20.3	77	90 (70–100)	81.5 ± 22.6	35	85 (75–100)	85.3 ± 13.8	n.s
Sport/play 5 y	107	80 (80–95)	76.0 ± 24.7	64	85 (75–95)	77.7 ± 26.5	43	75 (55–95)	73.5 ± 21.6	n.s
QoL 1 y	112	75 (63–94)	73.2 ± 23.2	77	81 (63–94)	75.0 ± 22.1	35	75 (50–94)	69.3 ± 25.3	n.s
QoL 5 y	107	75 (50–88)	67.4 ± 26.0	64	75 (53–92)	69.4 ± 27.0	43	63 (50–88)	64.4 ± 24.5	n.s
EQ-5D										
EQ-5D index 1 y	107	1 (1–1)	0.9 ± 0.8	74	1 (0.8–1)	0.9 ± 0.1	33	1 (0.8–1)	0.9 ± 0.1	n.s
EQ-5D index 5 y	107	0.8 (0.8–1)	0.9 ± 0.2	64	1 (0.8–1)	0.9 ± 0.2	43	0.8 (0.7–1)	0.9 ± 0.1	n.s
EQ-5D VAS 1 y	102	91 (80–99)	87.7 ± 14.1	72	95 (81–100)	89.4 ± 12.8	30	88 (80–96)	83.6 ± 16.4	n.s
EQ-5D VAS 5 y	106	83.3 (70–94	78.2 ± 20.7	63	89 (74–95)	79.3 ± 23.3	43	79 (70–87)	76.6 ± 16.4	n.s

KOOS and EQ-5D presented for 1 and 5 years (y) after ACLR

Age at ACLR, sex and time from injury to ACLR did not correlate with the outcome of the KOOS and EQ5D at 1, 2 and 5 years, except for sex on the KOOS ADL and knee-related QoL 5 years postoperatively. The mean value 5 years postoperatively for the KOOS ADL was 74.4 for females and 79.4 for males and, for the KOOS QoL, 64.5 and 73.7 for females and males respectively.

A significantly lower score was seen in revised patients on the KOOS subscales of Sports and recreational activities and knee-related QoL 5 years postoperatively. The mean value for Sports and recreational activities was 63.0 for revised patients and 78.1 for non-revised (*p* = 0.021). Revised patients reported a mean value of 45.8 and non-revised 70.9 for QoL (*p* = 0.001). No difference was seen between revised and non-revised patients on any of the remaining subscales 5 years postoperatively, or on any of the subscales 1 and 2 years postoperatively.

### Rehabilitation and return to sports

All patients at center A did not participate in sports until 9 months or more after ACLR. Sixty-eight percent of the patients (*n* = 52) at center B were, according to the medical record, allowed to return to the desired activity level including pivoting activities before 9 months post-operatively (34 patients at 6 months and 18 at 7–8 months). Twenty-six percent (*n* = 20) returned to sports at 9 months or later due delayed recovery of knee function and knee muscle strength, sometimes in combination with recurrent knee problems such as pain, swelling, and/or decreased ROM. Reliable information was missing in three cases, and in two cases there was a new knee trauma without relation to sports at 5 months. Consequently, the rehabilitation period was prolonged in these two latter cases.

Surgical center was, after multivariable logistic regression analysis, the only variable associated with revision rate (*p* = 0.019) (Table 4). Eight percent (9 of 116) of the patients treated at A (bracing with restrictive ROM and delayed return to sports) and 19% (15 of 77) of the patients operated on at B (free mobilization postoperatively with an early return to sports) underwent ACL revision (Table 2).

**Table 4:** Multivariable logistic regression analysis

	*B*	S.E	Wald	*df*	*p*	OR	95% CI for OR	
							Lower	Upper
Variables in the equation								
Surgical center	1.06	0.451	5.49	1	0.019	2.876	1.189	6.96
Constant	− 2.48	0.347	50.88	1	< 0.001	0.084	N/A	N/A
Variables not in the equation								
Age at ACLR^b^	N/A^a^	N/A	0.46	1	0.499	N/A	N/A	N/A
Sex	N/A	N/A	2.41	1	0.120	N/A	N/A	N/A
Time from injury to ACLR^c^	N/A	N/A	1.93	1	0.165	N/A	N/A	N/A
Graft diameter^d^	N/A	N/A	0.66	1	0.416	N/A	N/A	N/A

^a^N/A = Not Applicable

^b^Dichotomized above 13 years (yes/no)

^c^Dichotomized more than 5 months (yes/no)

^d^Dichotomized smaller than 8 mm (yes/no)

Comparisons between revised and non-revised patients regarding sex, age at ACLR, time from injury to ACLR, and graft diameter are described in Table 5.

**Table 5:** Non-revised vs revised patients

Variabels	Non-revised patients (*n* = 169)	Revised patients (*n* = 24)	*p* value
Median age at ACLR, years^a^	14 (13–14)	14 (13–14)	0.217
Female, *n* (%)	99 (58.6)	19 (79.2)	0.053
Median time from injury to ACLR, months^b^	6 (4–10)	3 (2–7)	0.059
Mean graft diameter, mm^c^	8,1 ± 0.8 (6–10)	8,1 ± 0.7 (7–10)	0.784

Values are expressed as mean ± SD (minimum–maximum) or as median (25th–75th percentile) when not normally distributed

^a^Dichotomized above 13 years (yes/no) in statistical analysis

^b^Dichotomized more than 5 months (yes/no) in statistical analysis

^c^Dichotomized smaller than 8 mm (yes/no) in statistical analysis

## Discussion

The most important finding was an overall revision rate at 12% with a lower rate of revision in patients operated at a center which applied a more restrictive postoperative rehabilitation protocol.

There was a large variation in the literature regarding ACL revision and graft failure rates. In a long-term follow-up by Reid et al., a revision rate of 9% was seen in patients younger than 16 years of age at the time of ACLR [24], which is similar to the results in a recent review by Zacharias et al., where graft failures occurred in 8.3% in a skeletally immature patients [25]. In a Danish registry study, the revision rate in the age group of 13–15 years was 6.7% [26]. In a review by Morvan et al., a re-rupture rate of 16% was seen in patients under 16 years of age [27]. Ho et al. found graft failure rates of 17% in skeletally immature patients, when graft failures were confirmed by clinical examination and magnetic resonance imaging, or by ACL revision surgery [11]. A rupture rate of 28% was reported among male patients younger than 18 years, compared with 14% in males older than 18 years [12].

As much as one third of the ACL reconstructed knees needed further surgery, and a contralateral ACLR was performed in 12% of the patients. This is important to consider when planning for ACLR in children and adolescents. This can be compared to the results by Lord et al., where one sixth of the ACL reconstructed knees in adults required further surgery, although the follow up time was only 2 years [28].

The overall meniscal injury rates of 31% for lateral meniscal injuries and 25% for medial meniscal injuries at the time of ACLR were slightly lower than the rates reported in previous studies of children and adolescents with ACL injuries. Vavken et al. reported 35% with lateral meniscal tears and 32% with medial meniscal tears [29]. Zoller et al. found lateral tears at the time of ACLR in 51% of the cases and medial meniscal injuries in 33% [9]. The presence of meniscal injuries differed significantly between the two centers, with a higher number reported in Group A for both lateral and medial meniscal injuries.

Several studies show a correlation between an increased time from injury to ACLR and higher rate of meniscal injuries in both children and adults [9, 30, 31]. The median waiting time from injury to ACLR was significantly lower at B; 5 months at Center B compared with 7 months at A. However, it can be questioned whether the time difference can fully explain the large difference in meniscal injuries. One possible explanation for the difference between the centers might be a dissimilar classification of small meniscal injuries. The meniscal injuries were repaired more often in Group A, whereas partial resection was more common in Group B.

The tibial fixation differed between the two centers. Patel et al. concluded in a review that the choice of tibial fixation technique remained controversial, and present clinical data did not demonstrate significant differences in patient outcomes or failure rates among methods [32].

The hamstring graft diameter was reported to predict graft failure. A graft diameter less than 8 mm increased the failure rate [13, 33]. However, in a recent study by Inderhaug, graft diameter was not found to be a risk factor for ACL revision surgery [34], nor did the present study confirm the association between graft diameter and revision rate. The mean graft diameter was 8.2 mm in Group A and 7.9 mm in Group B, respectively. Non-revised and revised patients had similar mean graft diameter.

The two study groups differed significantly in their distribution of age at ACLR from younger patients in Group A, and in the female:male ratio, with more females in Group B, but neither age at ACLR nor sex was a statistically significant confounding factor when analyzed in multivariable regression analysis. The difference in age might be explained by the fact that Hospital A is a purely pediatric hospital. It was more obvious for the parents of the youngest patients to present themselves to this kind of healthcare unit.

A higher failure rate for the skeletally immature population was seen in the study by Ho et al. [11]. The failure rate among patients with open growth plates was 17% compared with skeletally mature patients who had a failure rate of 8.6%. In the review by Wiggins et al., the authors stated that young age was strongly associated with a secondary ACL injury, either an ipsilateral re-injury or a contralateral ACL injury, although age was most frequently defined as under 18 or 20 years of age [35].

In a systematic review and meta-analysis by Tan et al., the large majority of the studies of graft rupture/failure reported no significant sex difference. However, there was a slightly higher relative risk for females compared with males regarding revision surgery (RR: 1.15 (95% CI 1.02–1.28)) [6]. In a recent review by Zacharias et al., no significant difference was seen in graft failure between male and female pediatric and adolescent patients [25].

There was no significant difference in the KOOS or EQ5D between the two postoperative rehab protocols at 1 year after ACLR. For this reason, bracing did not appear negatively to affect subjective knee function after surgery. The patients’ knee function at 1 year after surgery corresponded to what was reported in adults 2 years after ACLR [36]. The change from 1 to 5 years postoperatively did not show differences larger than the minimally important change (MIC), except for sport and recreation, where the score was lower at 5 years compared with 1 year postoperatively. The MIC for the KOOS was suggested to be 8–10 points, although the MIC is dependent on several factors [23]. Revised patients scored lower on the KOOS subscales of Sports and recreation and QoL 5 years postoperatively compared with non-revised patients. The difference was larger than the MIC, which indicates that a re-rupture and the following revision caused a serious impact on the subjective knee function. This was in accordance with the findings in adults by Cristiani et al. [37].

In this long-term follow-up study of 193 children after ACLR, we found that the application of more restrictive postoperative management, including the use of a postoperative brace with limited ROM, partial WB and a minimum of 9 months before returning to sports, was related to a significantly lower ACL revision rate.

The ACL graft undergoes ligamentization with a gradual increase in collagen, similar to what was seen in the native ACL, as shown by Amiel et al. in 1986 [38]. Further biomechanical research on ACL autografts in living patients showed that the total collagen content was relatively lower in hamstring grafts than in the native ACL 4–6 months after ACLR and it increased significantly in the period of 5–7 months postoperatively. Eleven to 13 months after ACLR, the collagen content was comparable to that of a native ACL [39].

Rodeo et al. showed that the healing of tendon to bone in a drill channel in dogs was most intense during the first 4 weeks postoperatively and it took 8–12 weeks for the tendon tissue to heal completely into the wall of the bone channel [40]. It is of course impossible from the results of the present study to separate the impact of the brace and the delayed return to sports respectively when it comes to the above-mentioned biological healing processes. However, 33% of the revisions in Group A and 40% in Group B of the re-injuries occurred during the first postoperative year, when the risk would be expected to be highest due to ligamentization. In a recent study by de Francesco et al., almost half the re-tears in ACL-reconstructed pediatric patients occurred before clearance to return to full activity. The first re-tears occurred 6 months after surgery and rose dramatically at around 9–12 months. Pediatric patients’ difficulty adhering to postoperative restrictions was suggested as one possible factor in early re-ruptures [41]. It might be the case that a postoperative brace had a positive impact on tendon-to-bone healing but also regarding compliance with a rehabilitation program, including a delayed return to sports. However, further research on this is needed to support this assumption.

The need for defined criteria before return to sports was emphasized in a review by van Melick et al. In cases of pivoting or contact sports, muscle performance and jump tests were recommended to be 100% compared with the uninjured leg and the return to sports should not implemented until 9 months postoperatively [42]. In a systematic review and meta-analysis by Wiggins et al., the return to a high activity level was also a factor strongly associated with a new ACL injury, either an ipsilateral rupture of the ACL graft or a contralateral ACL injury [35]. Despite the fact that center B had a more intensive and faster rehabilitation program, 26% of the patients waited until 9 months or more postoperatively before returning to the desired physical activity level. This might indicate that an early return to sports in the clinical setting, is not without complications and setbacks.

### Strengths and limitations

The strengths of the present study are the large number of patients, the minimum five-year follow-up period and the use of structured treatment protocols, but there are also several weaknesses. The patients in the two study groups differed in their characteristics. Consequently, the results of the comparison need to be interpreted with caution.

The revision ACLR rate was used as a primary outcome as opposed to graft failure, which may underestimate the risk of re-injury. In the review by Wiggins et al., 9 of 19 studies included in the meta-analysis used surgery (ACL revision or ACLR on the contralateral knee) as the primary outcome measurement and 10 studies used injury as the primary outcome. In the only study in which all the patients were < 16 years of age, surgery was used as the primary outcome measurement [35].

As mentioned, previous studies stated that a return to pivoting activities was strongly associated with a secondary ACL injury [35, 43]. The lack of exact information regarding the type and level of sports activity before and after ACLR, as well as the exact time of return to sports, are limitations in the present study. The most common cause of injury in both groups regarding the index injury was soccer (42%). Soccer was also the most common cause of re-injury, which might indicate that the patients attempted to return to their previous activity levels. The rate of ACLR of the contralateral knee was similar at both centers and might also give an indication of the patients’ postoperative activity level.

Another weakness is the high loss of follow-up registrations regarding PROMs. In cases with new injuries, revision or bilateral surgery KOOS will reflect the current knee function in an intention to treat analysis. Further limitations are that the PROMs used in the SNKLR are not adjusted to children and young patients. In particular, 10- to 12-year-old patients have difficulty understanding the questions in the KOOS. An adjusted scale, KOOS child, has been developed [44]. However, we felt it was important to present the subjective knee function values available in our study, since they still give an indication of the patient’s subjective knee function, and have been used in numerous previous studies evaluating pediatric patients after ACL injury [45]. In future research, child-specific PROMs would be preferable.

## Conclusion

Further surgery in the operated knee could be expected in one third of the cases including a revision rate of 12% after ACLR in patients younger than 15 years of age. The study also disclosed a similar rate of contralateral ACLR at 12%. The revision rates following pediatric ACLR was lower in a center which applied a more restrictive rehabilitation protocol.

## Data Availability

Data and Material are available upon request from the corresponding author.
